# EEG oscillations and related brain generators of phonation phases in long utterances

**DOI:** 10.1038/s41598-025-13901-8

**Published:** 2025-08-09

**Authors:** Said-Iraj Hashemi, Guy Cheron, Didier Demolin, Ana Maria Cebolla

**Affiliations:** 1 Laboratory of Neurophysiology and Movement Biomechanics (LNMB), Faculty of Human Motor Sciences, Université́ Libre de Bruxelles, Brussels, Belgium; 2 Phonetics and Phonology Laboratory LPP, CNRS-UMR 7018, Sorbonne Nouvelle, Paris, France

**Keywords:** Brain rhythms, Prolonged-phonation, Subglottic pressure, Respiratory control, EEG, SwLoreta, Neuroscience, Physiology

## Abstract

While the role of brain rhythms in respiratory and speech motor control has been mainly explored during brief utterances, the specific involvement of brain rhythms in the transition of regulating subglottic pressure phases which are concomitant to specific muscle activation during prolonged phonation remains unexplored. This study investigates whether power spectral variations of the electroencephalogram brain rhythms are related specifically to prolonged phonation phases. High-density EEG and surface EMG were recorded in nineteen healthy participants while they repeatedly produced the syllable [pa] without taking a new breath, until reaching respiratory exhaustion. Aerodynamic, acoustic, and electrophysiological signals were analyzed to detect the brain areas involved in different phases of prolonged phonation. Each phase was defined by successive thoracic and abdominal muscle activity maintaining estimated subglottic pressure. The results showed significant changes in power spectrum, with desynchronization and synchronization in delta, theta, low-alpha, and high-alpha bands during transitions among the phases. Brain source analysis estimated that the first phase (P1), associated with vocal initiation and elastic rib cage recoil, involved frontal regions, suggesting a key role in voluntary phonation preparation. Subsequent phases (P2, P3, P4) showed multiband dynamics, engaging motor and premotor cortices, anterior cingulate, sensorimotor regions, thalamus, and cerebellum, indicating progressive adaptation and fine-tuning of respiratory and articulatory muscle control. Additionally, the involvement of temporal and insular regions in delta rhythm suggests a role in maintaining phonetic representation and preventing spontaneous verbal transformations. These findings provide new insights into the mechanisms and brain regions involved in prolonged phonation. These findings pave the way for applications in vocal brain-machine interfaces, clinical biofeedback for respiratory and vocal disorders, and the development of more ecologically valid paradigms in speech neuroscience.

**Supplementary Information:**

The online version contains supplementary material available at 10.1038/s41598-025-13901-8.

## Introduction

The production of speech in humans relies on the precise integration of respiratory cycles, the articulatory system, and the regulation of subglottic pressure (Ps). Titze (1994)^1^ proposed that each respiratory cycle for phonation comprises an inspiration phase and three expiration sub-phases (initial active, passive, prolonged active). The neural networks involved are widely distributed across the pontomedullary brainstem. Specific premotor areas are associated with different aspects of the post-inspiratory period, such as the pre-Bötzinger complex and the adjacent Bötzinger complex. These areas interact to generate the three-phase respiratory motor pattern. The pontine Kölliker-Fuse nucleus is involved in the transition from the inspiratory to the post-inspiratory phase^2,3^ (P. Trevizan-Baú et al. 2024, for a detailed revision^4^. For short utterances, the elastic recoil of the inspiratory muscles is sufficient to maintain Ps; for long utterances, tonic contraction of the thoracic and abdominal expiratory muscles (internal intercostals, external obliques, rectus abdominis) is necessary^5,6^. Ladefoged et al. (1967) showed that Ps is regulated passively during a long statement by the elastic recoil of the lungs and the external intercostal muscle and then through the tonic contraction of the internal intercostal, external oblique and rectus abdominis muscles^7–9^.

Beyond these mechanisms localized at the muscular and brainstem levels, the rhythmic dimension that governs speech has also been investigated. Human speech involves rhythmic synchronization in the theta band, which matches the syllable tempo created by jaw movements and vocal foldvibrations^10–13^. Interestingly, it has been shown that marmoset monkeys exhibit vocalizations with coupled phono-articulatory oscillations that are synchronized and phase-locked at theta rhythms suggesting that such phono-articulatory theta oscillation may constitute an intrinsic mechanism already present in primate vocal production^13^. Although consistent evidence is growing^14–18^it remains to be confirmed whether such theta rhythmicity and speech-breathing synchronization are controlled by subcortical and or cortical entities^13–18^.

To resolve this ambiguity, neuroimaging studies have examined brain engagement during phonation. fMRI findings have shown that compared to controlled expiration through the nose, sustained vocalization in healthy adults is accompanied by activation of bilateral auditory cortex, dorsal and ventral laryngeal motor areas, cerebellum, insula, dorsomedial prefrontal cortex (dmPFC), amygdala, and periaqueductal grey region, and that significantly increased functional connectivity exists between the periaqueductal grey region and left ventral laryngeal motor areas during voicing^19^. The involvement of the left sensorimotor cortex and supplementary motor area has also been shown during short duration phonation and voluntary expiration^20^. Belyk et al. (2021)^21^ precisely localized the dorsal and ventral laryngeal motor cortices (dLMC and vLMC) and showed that both regions are robustly engaged not only during vocal fold vibration (humming) but also during respiratory control (whistling), highlighting their dual role in regulating Ps. Moreover, Kryshtopava et al. (2017)^22^ compared brain activation in women with muscle tension dysphonia to healthy controls and found hyperactivation of laryngeal motor areas alongside hypoactivation of sensory–auditory regions in patients, suggesting that an imbalance between motor drive and sensory feedback disrupts appropriate Ps regulation.

Nevertheless, fMRI remains limited in tracking the rapid dynamics underlying these interactions. Thanks to its temporal resolution on the order of tens of milliseconds^23^electroencephalography (EEG) allows direct capture of cerebral oscillations linked to the phonation. More concretely, the analysis of event-related spectral perturbations (ERSP) of EEG oscillations (ERSP measure, Delorme and Makeig, 2002)^24^ can elucidate the specific spectral behavior of brain oscillations linked to each phase of phonation, highlighting specific cerebral reactivities. For instance, Sörös et al. (2024)^25^ reported an atypical beta-band (16–31 Hz) synchronization, manifested as an abnormal increase in spectral power between 200 and 400 ms following a cue instructing participants to produce the syllable [papapa]. This aberrant beta ERS suggests altered oscillatory dynamics during the preparatory phase of speech production. Furthermore, oscillatory analyses have revealed a functional dissociation between alpha and beta rhythms within the sensorimotor cortex. Intracranial recordings have shown that alpha-band ERD (8–13 Hz) peaks during the somatosensory feedback phase, indicating sensory processing, whereas beta-band ERD (15–25 Hz) is sustained throughout the planning and motor execution of phonation, reflecting continuous motor system engagement^26^.

Furthermore, the respiratory cycles themselves appear to modulate these cortical oscillations. This is supported by growing evidence of a functional correlation between the breathing cycle and intracortical EEG (iEEG/gamma-band envelope) oscillations^27^. Distinctive neural networks in grey matter, assessed by iEEG in epileptic patients, have been described during volitional control of breathing. These networks include the caudal-medial frontal, premotor, orbitofrontal, and motor cortex, insula, superior temporal gyrus, and amygdala. During attention focused on one’s own breathing, the anterior cingulum, premotor, insula, and hippocampus are involved^27,28^. More recently, neural signatures of voluntary respiration have been demonstrated with scalp recorded EEG. When breathing voluntarily, the delta band (0–2 Hz) EEG power spectrum is significantly enhanced in frontal and right parietal scalp areas, and the sample entropy value of EEG decreases indicating a more orderly state. Also, there’s a positive correlation between respiration intensity (assessed by chest motion variations) and delta EEG-respiration phase-locking factor^29^. Yet very few studies have specifically addressed how these respiratory rhythms interact with prolonged phonation^20,22^.

In the present study, we investigate power spectral variations of EEG brain rhythms during prolonged phonation. We hypothesize that each transition between consecutive phases of phonation is accompanied by identifiable brain oscillatory reactivity. To test this, we developed a prolonged phonation paradigm inspired on that of Ladefoged et al. (1967) where participants continuously repeat the syllable “pa” without breathing for as long as possible. In addition, the generators accounting for such brain reactivities to prolonged phonation phases are estimated by a distributed solution (swLORETA).

## Methods

### Participants

Nineteen native French speakers, right-handed, healthy subjects (10 males, 9 females, 24 ± 6 years old) without neurological, psychiatric or respiratory history participated in this study. None of the participants had received formal training in singing or drama. All were native French speakers from the Brussels region. All participants gave informed consent to the experimental procedures. The study was approved by the Ethics Committee of the Brugmann University Hospital, Brussels (Ref. CE 2022/246). All methods were performed in accordance with the relevant guidelines and regulations.

### Design

The participants arrived at the Laboratory of Neurophysiology and Movement Biomechanics (LNMB, Université Libre de Bruxelles), received instructions for the experiment, and signed an informed consent form. After being equipped with an EEG cap, surface EMGs, and aerodynamic signal captors, participants sat comfortably in a chair and were asked to keep their eyes closed during the task. The protocol consisted of two tasks. In the first task, participants remained seated comfortably with their eyes closed, without moving and staying calm. In the second task, participants, still with their eyes closed, were asked to take a deep breath and then pronounce the syllable [pa] in a self-paced manner, as long as possible until exhaustion without taking a new breath. The experimenter gave the instruction to speak normally, and the start signal for each trial. Participants took a break at the end of each trial.

The syllable [pa] was chosen for its articulatory simplicity and physiological relevance. The bilabial plosive [p] involves complete closure of the lips, resulting in a build-up of intra-oral pressure which is released during production of the vowel [a]. This configuration provides a reliable, non-invasive approximation of Ps, as the glottis remains open during [p], allowing the pressure to reflect the total volume of the lungs and vocal tract.

^30^. Compared to other sounds that may be affected by nasalization or complex tongue movements, the production of the syllable [pa] is relatively easy to produce and highly reproducible from trial to trial and participant to participant.

^30,31^. Thus, its continuous production without breathing offers a practical and consistent method for tracking Ps dynamics over time in a non-invasive manner.

**Fig. 1:**
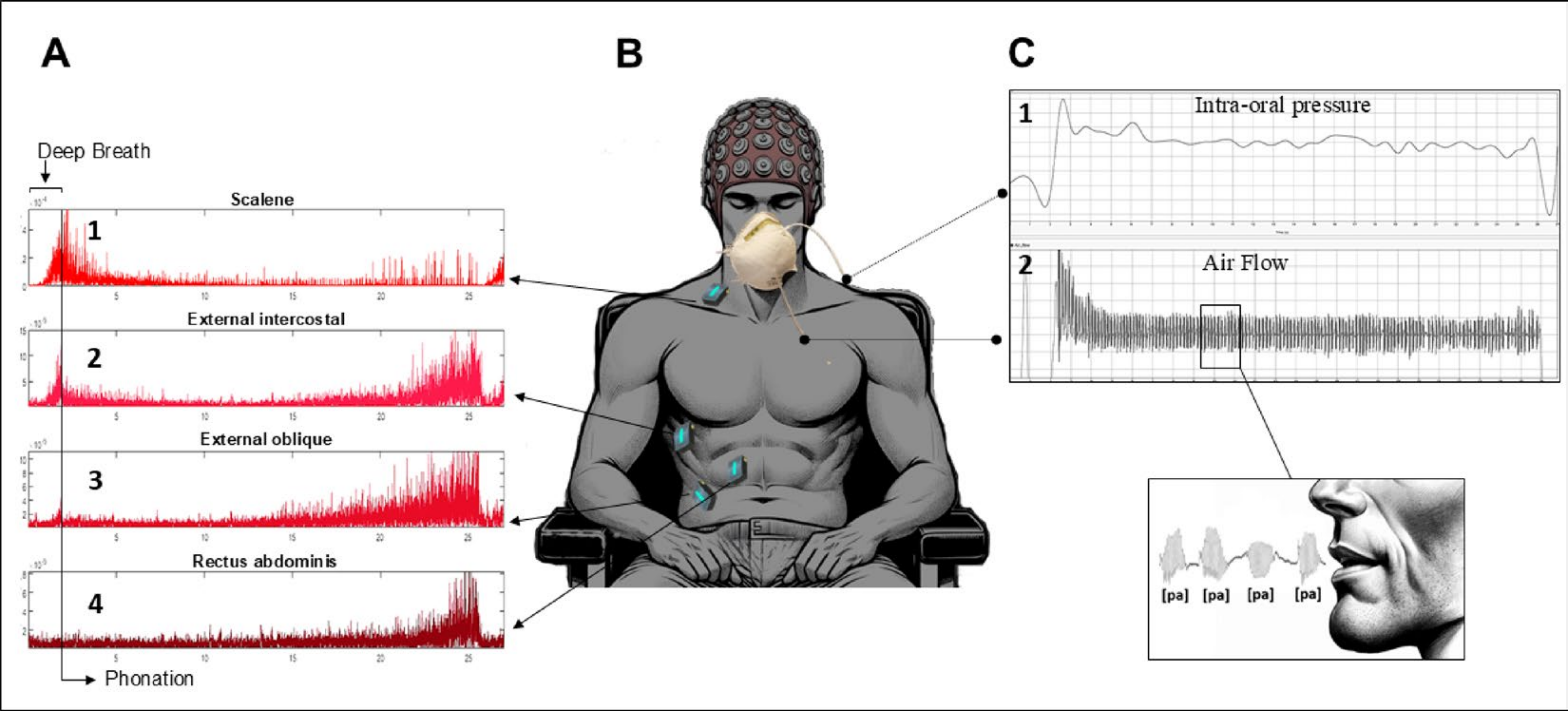
Experimental design. (**A**) Electromyography signals recorded by surface EMG sensors placed superficially on respiratory muscles. The vertical line on the left of image A indicates in the EMG signals, the end of inspiration and the beginning of the syllable production sequence. **1** Scalene EMG; **2** External intercostal EMG; **3** External oblique EMG; **4** Rectus abdominis EMG, over time. (**B**) EEG cap comfortably adjusted in the participant’s head for measuring brain activity by 64 EEG channels. (**C**) Experimental setup for aerodynamic signals measurements comprising two tubes going through a face mask. One of the tubes, is inserted into the subject’s mouth to measure intra-oral pressure which allows to obtain an indirect measure of Ps at the peak values of [p], while the other one is situated on the mask surface for collectting the airflow, during pronunciation of the syllable [pa]. **C1** Intra-oral pressure and **C2** Air flow signals.

#### EEG, EMG, and aerodynamic signal acquisition

EEG signals were recorded using the ANT Neuro eego™ sport system (ANT Neuro system, The Netherlands) at a sampling frequency rate of 2048 Hz. An active-shield cap with 64 Ag/AgCl-sintered ring electrodes (following the 10–20 electrode system placements) and shielded co-axial cables was comfortably adjusted to each participant’s head (Fig. 1B). The reference electrode was integrated into the EEG cap (Cpz). The impedance for the EEG electrodes was kept below 5 kΩ.

For the recording of electrical muscle activity, bipolar surface EMG electrodes from Delsys Trigno™ were superficially placed over the skin of the inspiratory scalene and external intercostal muscles, and the expiratory external oblique and rectus abdominis muscles^32,33^. To ensure optimal placement, the sensors were positioned while the subjects stood upright. All sensors were placed on the right side of the participants. Before electrode placement, the participant’ skin was cleaned with 75% isopropyl alcohol at the electrode sites. The sensor measuring scalene activity was placed in the posterior triangle of the neck, at the level of the cricoid cartilage. The sensor for the external intercostal muscle was positioned between the 7th and 8th ribs along the axillary line. For the external oblique muscle, the sensor was placed on the right side, from the center of the muscle, 3–4 cm from the navel, in a diagonal direction. Finally, the rectus abdominis sensor was placed just above the navel on the right side^34–36^. All sensors were aligned parallel to the muscle fibers^37^. The EMG signals were collected at a sampling rate of 2000 Hz (Fig. 1A).

Participants were continuously monitored throughout the trials to quickly detect any abnormal behavior or movement. Episodes of swallowing or torso turning, identified visually, led to the exclusion of the corresponding trials. To prevent muscle fatigue, subjects took a 5 to 10-second break between each trial and could request a longer delay if needed; however, none required additional breaks.

The aerodynamic signals were recorded using an AeroMask dispositive^38^ composed of two tubes connected to a face mask. One tube, for the intra-oral pressure measurement, went through the mask and was placed inside the subject’s mouth, without causing discomfort, The other one was attached to the surface of the mask for the airflow measurements. These aerodynamic signals were collected at a sampling rate of 8000 Hz (Fig. 1C). During the production of a stop consonant followed by a vowel, such as [pa], intra-oral pressure increases during the lips closure and drops sharply when they open. The peak of intra-oral pressure reached during the bilabial closure is a good indirect indicator of Ps.

EEG signals, surface electromyography (EMG), and aerodynamic data (airflow and intra-oral pressure) were synchronously recorded using an external Rigol DG1022 20 MHz signal generator, sending triggers at the start and end of each recording to the three recording systems. During off-line processing, any data collected before the triggers was discarded, aligning in this way the temporal starting point (time 0) of all data from the different recording systems.

#### Signal processing

Off-line data treatment was conducted using MATLAB (The MathWorks, Natick, MA, USA; version R2021a) and the EEGlab toolbox. For the estimations of the brain generators from EEG signals, swLORETA model (standardized low-resolution weighted electromagnetic tomography) is available in Advanced Source Analysis software (ANT neuro).

#### EMG and aerodynamic signal processing

##### Phases of phonation

Titze (1994) proposed that one breathing cycle of respiration in a long phonation sequence can be divided into one inspiration phase and one expiration phase, which itself can be subdivided into three sub-phases: (1) the elastic recoil of the lungs and external intercostal muscles followed, in sequence, by (2) the activation inner intercostal and (3) external oblique muscles. This last phase is divided into 2 parts in our work: the activation of the external oblique muscles and rectus abdominis (4). The boundaries of the different phases were determined from the analysis of the EMG signal, notably by calculating its second derivative and intra-oral pressure. Prior to this step, preliminary data processing was performed. The Ps signal was low-pass filtered using a 6th-order (to enhance cutoff sharpness) Butterworth filter with a cutoff frequency of 70 Hz to remove high-frequency components associated with glottal vibrations and retain only the slow pressure envelope related to Ps. To avoid temporal distortion, the filter was applied in a zero-phase manner using the filtfilt method^39,40^. For EMG signals, the signal-to-noise ratio (SNR) across muscles ranged from 13.5 dB to 18 dB. EMG data were band-pass filtered using a 6th-order Butterworth filter with a high-pass cutoff at 20 Hz to attenuate motion artifacts and low-frequency noise, and a low-pass cutoff at 400 Hz to suppress high-frequency noise while preserving the relevant spectral content of muscle activity^41,42^. As with the Ps signal, filters were applied using zero-phase filtering. All signals (Ps and EMG from the external oblique, rectus abdominis, and external intercostal muscles) were then converted into a peak upper envelope using a 300 ms sliding window to capture amplitude trends. This window length corresponds to the average syllable duration plus one standard deviation. For EMG signals, after filtering, baseline noise was estimated for each channel during a 2-second pre-phonation rest period. An activation threshold was defined as the mean baseline level plus three standard deviations (mean + 3·σ), ensuring that 99.9% of resting noise remained below threshold. This threshold was computed individually for each participant and each muscle.

To identify muscle activation and deactivation points, we adopted a dual-approach strategy. The first method, referred to as the “Peak Upper” approach, involved computing the amplitude envelope using the 300 ms sliding window described above. This allowed for rapid visual detection of threshold crossings based on the rectified EMG signal. A first visual inspection was performed by the primary examiner, and a second independent verification was conducted by another experimenter to ensure consistency. The second method consisted of an automated verification using the second derivative of the Hilbert-transformed analytic envelope, which was subsequently low-pass filtered at 40 Hz to suppress high-frequency noise. Both methods used the same activation threshold (mean + 3·σ) and applied the same minimum duration criterion (≥ 2 s), ensuring consistency and comparability. Manual visual inspection remains the clinical gold standard for identifying EMG activation patterns, while the Hilbert-based method provides a more objective and reproducible measure of the same events.

For the external oblique and rectus abdominis muscles, activation onset was defined as the first point where the envelope crossed the threshold with a positive second derivative and remained above threshold for at least 2 s, to exclude brief phasic bursts. Deactivation was marked at the end of phonation, when the envelope fell below threshold. For the external intercostal muscle, which is primarily recruited during the preceding inspiration, the onset was similarly defined by a sustained suprathreshold crossing (≥ 2 s), while offset was identified as the first subsequent crossing below threshold, accompanied by a negative second derivative.

This calculation was performed for each trial, and consistency was verified by examining the concomitance between these inflection points and the transient variations in the estimated intra-oral pressure. It should be noted that the inflection points of the pressure and the EMG show a slight time lag (50 to 200 ms), characteristic of the physiological delays between intra-oral pressure and the muscular response^43,44^.

**Fig. 2:**
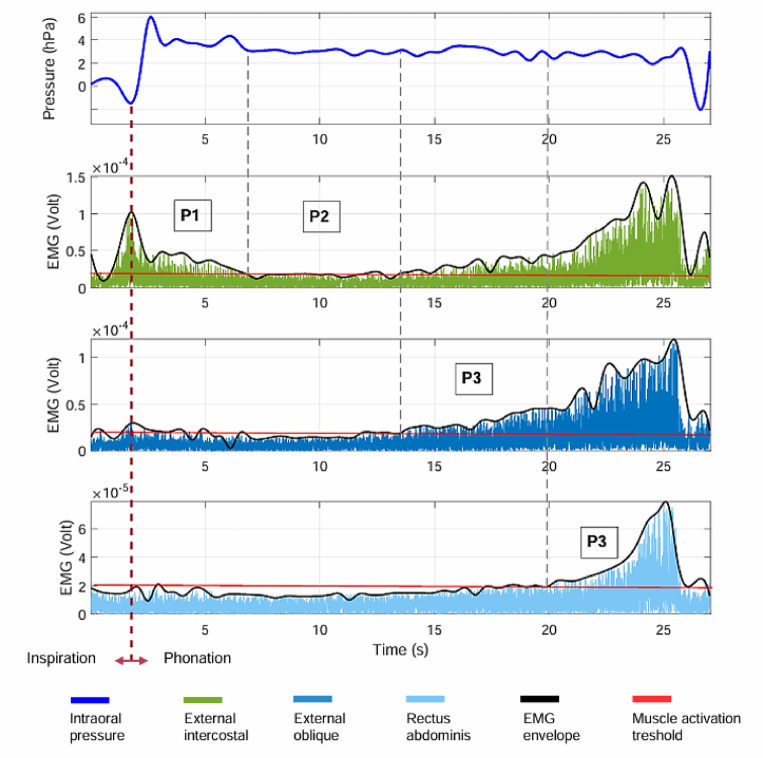
Phases (P1, P2, P3 and P4) of long utterance phonation determined by changes in respiratory muscle activity during the production of syllable [pa]. The curves represent a single trial.

**Fig. 3:**
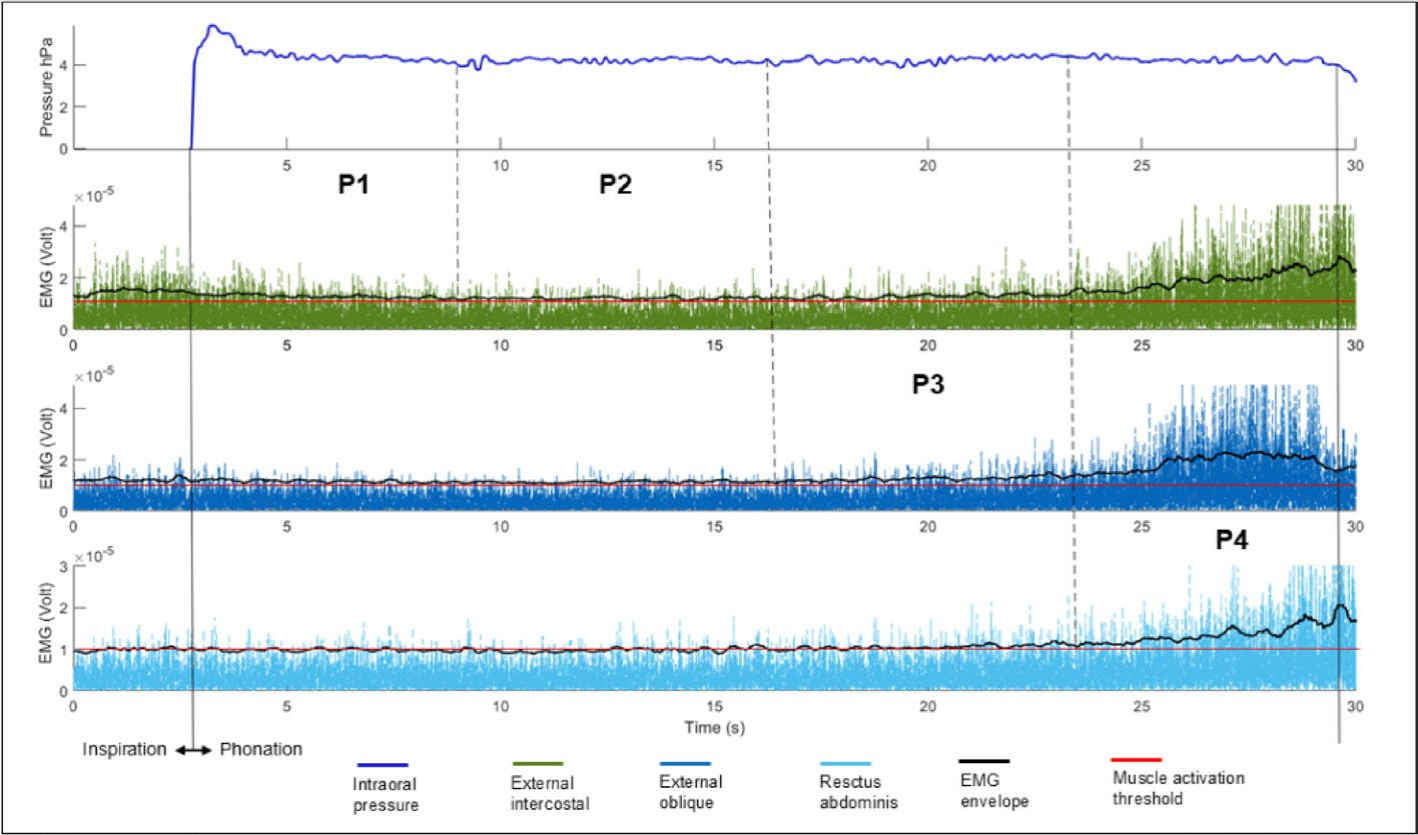
Phases (P1, P2, P3 and P4) of long utterance phonation determined by changes in respiratory muscle activity during the production of syllable [pa]. The curves represent an average of 120 trials of 10 subjects.

Phase 1: The period between a maximal peak of Ps marking the transition from inspiration to phonation (event phase 1), and the subsequent inflection point where Ps reaches a minimum, which coincides with a similar inflection in external intercostal muscle activity, reflecting the end of elastic recoil.

Phase 2: The period between the preceding minimal point of inflection characterizing Phase 1’s end, and the starts of External Oblique muscle activation (point of inflection), (event phase 2).

During this phase, there is no notable variation in intra-oral pressure. It has been previously shown that the activity of the internal intercostals (not recorded here) is considerable during this phase^32,34^.

Phase 3: The period between the concomitant inflection points characterizing Phase 2’s end and the inflection point in the rectus abdominis EMG trace indicating its activation (event phase 3). Note an increase in the activity of the external intercostals towards the end of this phase.

Phase 4: the period between the minimal inflection point in the rectus abdominis EMG trace characterizing Phase 3’s end concomitant to a maximal inflection point indicating in the intra-oral pressure decrease till the end of phonation (event phase 4).

Transition: After phase 4, there is a break before the next phonation sequence.

#### EEG signal processing

Data processing and statistics were performed using EEGLAB software^45^ (version v2021.1). The first step in the offline EEG processing involved importing the previously determined temporal events into the EEG trials. These events, which include the trial onset, the onset of each respiratory phase, and the trial end, indicate the start of each phase in the four phases of respiratory muscle activation. These temporal events were determined on a trial-by-trial basis for each participant (Figs. 2 and 3).

In the preliminary EEG data treatment, a low-pass filter of 200 Hz, a resampling to 512 Hz, and a high-pass filter of 0.5 Hz were applied. Zapline plugin^46^ was used for removing artefactual 50 Hz coming from the alternating current interference. Then, all artificial portions of the EEG data were rejected through visual inspection. EEG signals were re-referenced to REST (Reference Electrode Standardization Technique)^47^. Synchronous or partially synchronous artificial activity (mainly blinks and muscle activities) was detected and rejected by Independent Component Analysis (ICA) on continuous data. Following this, the Artifact Subspace Reconstruction (ASR) algorithm^48–50^ was used. Unlike ICA, ASR performs with a non-stationary method to correct artifacts. ASR relies on the recording of the rest condition to calculate the variance of the clean EEG signals. The chosen standard deviation (SD) threshold for ASR was between 20 and 100, so that a minimum of 80% of the original signal variance was preserved^51^.

We transformed the continuous EEG data into epochs extracted from − 2 to 4 s with respect to every event linked to each of the four phases. The choice of 4 s is justified by the fact that this duration represented the shortest duration among 95% of the phases.

After a final visual inspection of the epochs, a total of 325 epochs per phase remained. The baseline for phase 1 (-2 to 0 s) was defined by the 2 s preceding the start of the trial, while for subsequent phases, the baseline was determined by the last 2 s of the previous phase.

To assess the specific aspect of brain information processing that is time-locked to the events of interest (each of the four phases), we used the ERSP time-frequency analysis^52–54^. ERSP measures variations at specific periods in the power spectrum of ongoing rhythms that are induced by the event of interest. In ERSP measures, ERD (event-related desynchronization) indicates a reduction in the power spectrum, while ERS (event-related synchronization) indicates an increase in the power spectrum. ERD/ERS are interpreted as reflecting brain reactivity. Standard ERSP analysis^24^ is normalized with respect to the preceding time-period of the event of interest. This is of special interest for investigating the dynamic changes underlying the transition of one phase to the next one, as normalized ERSP allows to pop out the power spectrum variations specifically with respect to the preceding period. We used wavelet transformation for a complex spectro-temporal representation with Hanning windowed sinusoidal wavelets. ERSP patterns were calculated with 400 time points (-1500 to 3998 ms) at 100 linearly spaced frequencies from 1 to 45 Hz. For the significance level of ERSP, bootstrap resampling (*p* < 0.001) was used as a surrogate method, and a false discovery rate (FDR) correction was applied. Later, the statistical differences of the topographical ERSP (64 electrodes) between phases were calculated by permutation analysis (*p* < 0.05), with the Holms method (Holm, 1979; McFarland et al., 2000; de Lange et al., 2008) for the correction of multiple comparisons in steps of 500 milliseconds.

#### Source analysis

We estimated the brain sources using the standardized weighted Low Resolution Brain Electromagnetic Tomography (swLORETA) method (described in detail in Cebolla et al. 2011)^55^. Within the family of distributed methods, swLORETA allows for an accurate reconstruction of current sources at both surface and depth in simulated data, even in the presence of noise and when two dipoles are active simultaneously. This result is achieved by incorporating a singular value, a lead-field weighting based on decomposition that compensates for background noise and accounts for the variable sensitivity of sensors to current sources at different depths.

To map the generators of the main ERSP components of phases 1 to 4, we calculated the swLORETA sources solutions based on the ERSP individual topographies triggered by the phase changes, in the 4 s time intervals of every phase in steps of 1000ms (following Cebolla et al., 2011). As part of the swLORETA inverse solution analysis in the Advanced Source Analysis (ASA)^56^ software (version 4.10.1), the Boundary Element Model (BEM) was used to solve the direct problem. The inverse solution was limited to the gray matter based on the probabilistic maps of brain tissues available from the MNI^57^. The voxels (grid size of 10.00 mm) and the arrangement of electrodes were placed according to the Collins 27 MRI produced by the Montreal Neurological Institute^57^.

For the statistical maps, using in-house MATLAB base tools, the current density of each voxel of each participant and each condition (P1 to P4) was divided by the average current density value of all voxels of the same participant and condition. Inter- and intra-subject variability was thus mitigated by using normalized single-trial dipole measures and within-subject contrasts. We thus obtained a normalized inverse solution in which a voxel value greater than 1 indicates greater-than-average activity. In the next step, 2000 permutations were performed to obtain a population map of the normalized inverse solution for each condition. Family-wise error rate (FWER) was controlled using a maximal statistic permutation procedure (Nichols & Holmes, 2002): concretely 2000 sign-flip permutations were performed under a paired design. For each permutation, dependent-sample t-values were computed across all dipoles, and the maximum t-value was extracted. The empirical t-max distribution defined a corrected threshold at the 95th percentile, ensuring strict FWER control across all contrasts. Normality was assessed via Kolmogorov-Smirnov tests (α = 0.01), and homogeneity of variance by Levene’s test. Depending on these results, paired t-tests (parametric) or Wilcoxon signed-rank tests (non-parametric) were used.

To investigate the source dynamics of the phase transitions, we then calculated the contrasts P1 > baseline, P2 > P1, P3 > P2 and P4 > P3.

The final Talairach coordinates^58^ (directly accessible for each voxel in the ASA software) provided in the results section correspond to the maximum cluster values of the final statistical maps. In addition, the corresponding Brodmann areas within the cortical mantle are provided (talairach.org from Research Imaging Institute).

## Results

### Acoustic and aerodynamic analysis

**Fig. 4:**
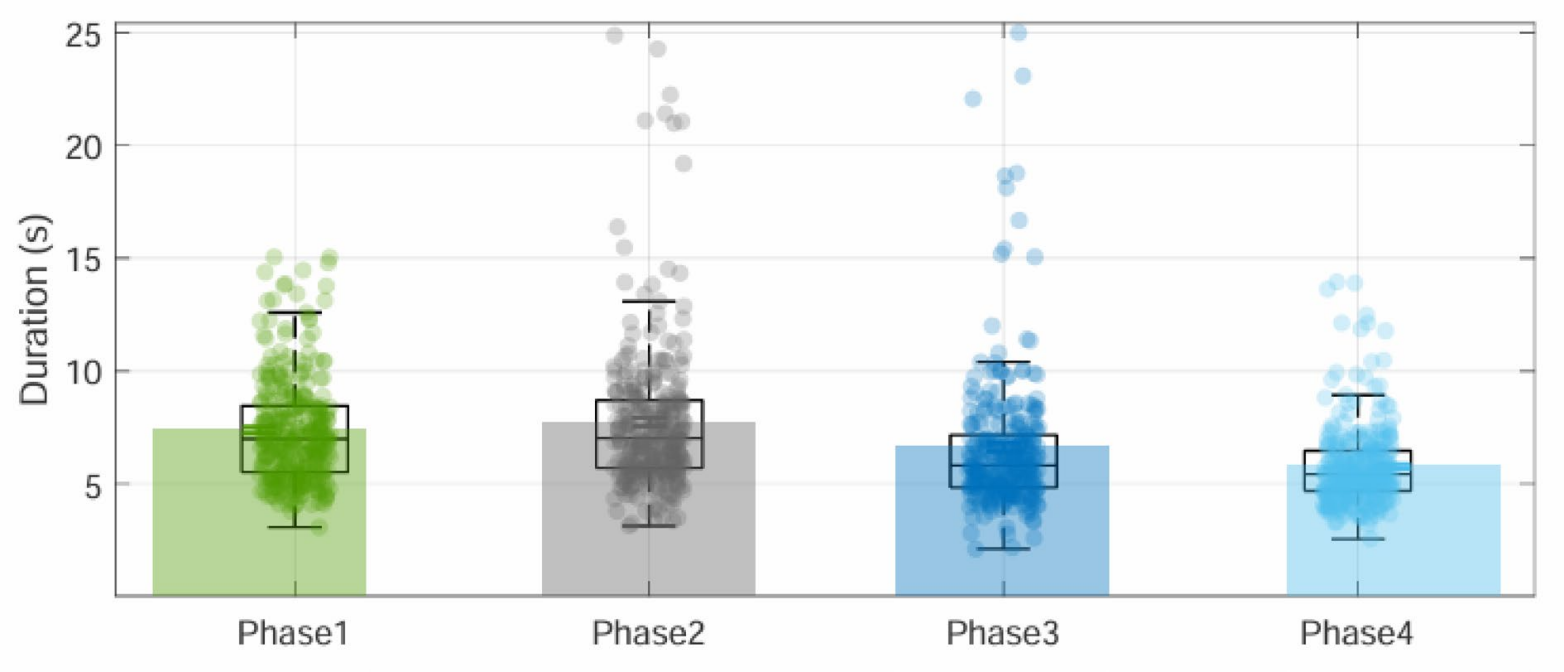
The variation of phase duration. Within each phase, each point represents a trial from a participant. Semi-transparent rectangles (green, gray, blue, and light blue) in the background denote the overall mean duration of each phase. Overlaid boxplots show, for each phase, the median marked by the horizontal line within the box, the first and third quartiles represented by the box edges, whiskers extending to the minimum and maximum observed values, and points beyond the whiskers indicating outliers.

The syllable [pa] was produced at an average rate of 4 ± 0.5 Hz, corresponding to a duration of 255 ± 46 ms. Phases P1 to P4 lasted on average 7.4 ± 2.3 s, 7.7 ± 3.1 s, 6.6 ± 3.1 s, and 5.8 ± 1.5 s, respectively. The mean trial duration was 27.5 ± 9 s, with trials exceeding 60 s for one participant. As shown in Fig. 4, the distribution of phase durations is broad for P1 to P3 but narrows for P4. This is reflected in the inter-subject standard deviations: 2.24 s (P1), 3.12 s (P2), 3.10 s (P3), and 1.51 s (P4). Intra-subject variability reveals trial-to-trial standard deviations of 1.08 s (P1), 1.57 s (P2), 1.56 s (P3), and 1.19 s (P4), indicating that P1 has the smallest intra-subject variability among the phases. These variability results are detailed in Supplementary Figure A1 and Table A1.

**Fig. 5:**
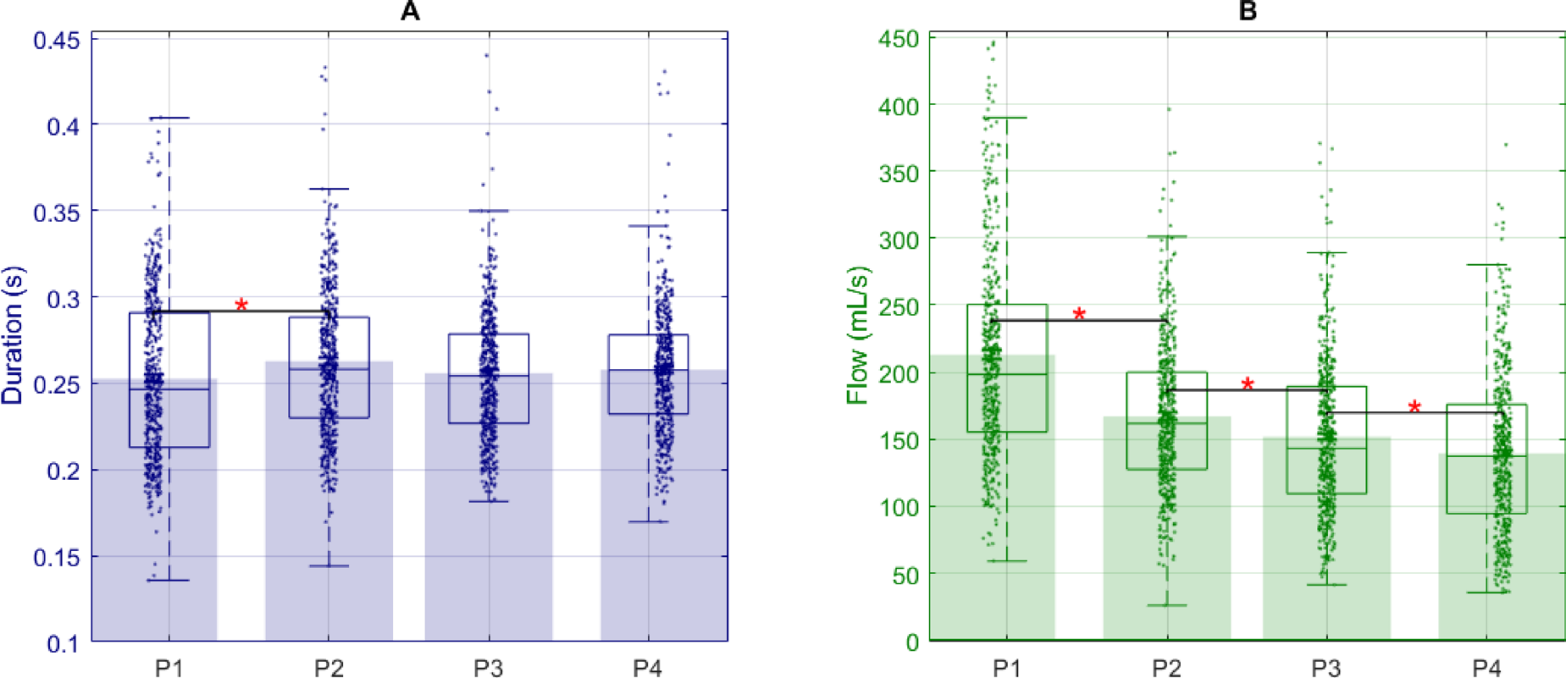
The variation of syllable [pa] duration and the associated airflow. (**A**) shows syllable duration, while (**B**) shows airflow. Semi-transparent rectangles (blue for syllable duration and green for airflow) in the background denote the overall mean duration and mean airflow. For both A and B, the boxplots show the median marked by the horizontal line within the box, the first and third quartiles represented by the box edges, whiskers extending to the minimum and maximum observed values, and points beyond the whiskers indicating outliers. For A and B, the asterisks mark significant phase differences.

For airflow per syllable and syllable duration by phase, tests for normality (Lilliefors) and homogeneity of variance (Levene’s) indicated that neither metric met these assumptions. Therefore, we applied Kruskal–Wallis tests with Dunn Sidak corrected pairwise comparisons throughout. Additionally, we calculated inter-subject and intra-subject variability for each of these two parameters. The duration of syllables [pa] was 253 ± 48 ms, 263 ± 49 ms, 256 ± 43 ms and 258 ± 39 ms in P1, P2, P3 and P4, respectively. For [pa] durations analysis significant differences between phases, we used the Kruskal-Wallis and the Dunn nonparametric post hoc test with a Bonferroni correction to make a pairwise comparison. Syllable duration in P1 was significantly shorter than in P2 (*p* < 0.05). There was no significant difference between the other phases (Fig. 5).

Similarly, we compared the airflow per syllable in each phase. The average airflow per syllable were 213 ± 83 ml/s, 167 ± 55 ml/s, 152 ± 57 ml/s and 139 ± 59 ml/s in P1, P2, P3 and P4, respectively. Each consecutive comparison (P1 > P2, P2 > P3, P3 > P4) was statistically significant at *p* < 0.05 (Fig. 5).

The intra-oral pressure was 4.52 ± 1.19 hPa, 4.11 ± 1.07 hPa, 4.18 ± 1.15 hPa, and 4.46 ± 1.7 hPa in P1, P2, P3, and P4, respectively. The intra-oral pressure in P1 was significantly higher than in P2 (*p* < 0.001), and it was significantly higher in P4 compared to P3 (*p* < 0.001). However, there was no significant pressure difference between P2 and P3 (*p* < 0.95).

### Time-frequency analysis of EEG

**Fig. 6:**
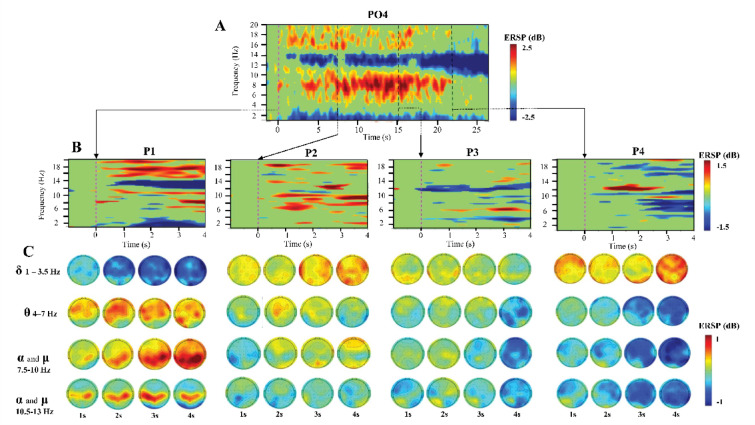
Grand average (Bootstrap *p* < 0.001 FDR) of the power spectral variations of the EEG oscillations during phonation. (**A**) ERSP templates for one representative electrode (PO4). (**B**) ERSP templates (PO4) for each phase. The latencies of the phonation phases are represented by bold, vertical red dotted lines (a bootstrap 0.001 was used). (**C**) Topographical distribution over the scalp for delta, theta, low, and high alpha rhythms, for each phase for successive periods of 1 s of duration. Vertical bold dotted lines: latencies of phases of phonation. For (**A**), (**B**) and (**C**), the baseline for phase 1 (-2 to 0 s) was defined by the 2 s preceding the start of the trial, while for subsequent phases, the baseline was determined by the last 2 s of the previous phase.

Figure 6A and B illustrates the grand average (19 participants) of the power spectral variations (bootstrap 0.001) of the EEG oscillations during phonation, by the ERSP templates for one representative electrode (PO4) and for their topographical distribution for the full array of electrodes (64) over the scalp (Fig. 6C). In the ERSP template (Fig. 6A), the power spectral variations for the four phases are measured with respect to the same period of 2 s preceding phonation onset, that is P1 at 0 ms. This illustrates the general rhythmical outcome induced by the constrained vocalization lasting throughout the four phases showing two long bands of power decrease or event-related desynchronization (ERD) at the delta (1–3.5 Hz range) and the high alpha (10.5–13 Hz range). These two ERD, a band of power increase or event related synchronization (ERS) more diffuse in between the low alpha (7.5–10 Hz range) and theta (4–7 Hz range) frequency oscillations appeared from the middle of P1 to the end of P3. Scattered short clusters in beta rhythms (15–20 Hz range) were observed from the start of P1 to the middle of P3.

To make pop out the power spectral variations induced by the transition between consecutive phases ERSP templates (Fig. 6B) were calculated separately for each phase (P1, P2, P3, P4) (for 4 s period) with respect to the last 2s period of the preceding phase. Note that while similar ERSP patterns appear in P1 of Fig. 6B as in corresponding P1 of Fig. 6A (congruent considering similar period of reference preceding P1), the ERSP templates for the other phases revealed new ERSP patterns specific to each phase transition.

In Fig. 6C the topographical distribution over the scalp for delta, theta, low and high alpha rhythms related to each phase are illustrated for successive periods of 1 s of duration. Interestingly, a gradual global topographical ERS/ERD inversion was observed across all four oscillation bands, becoming progressively more apparent throughout the different phases. This inversion was particularly pronounced when focusing on P1 and P4.

In a detailed manner, note that **P1** presented a progressive potentiation of delta ERD that expanded over the scalp through time, a persistent frontocentral ERS in the theta rhythm, a potentiation of postcentral and parietal ERS in the low alpha rhythm, and a consistent central high alpha ERS throughout the phase. **P2** initially presented delta ERS largely distributed over the scalp and being localized later bilaterally on temporal areas. Theta ERS was localized in frontocentral scalp areas at the 2 s. Low alpha power variations in P2 presented a well-delimitated ERD cluster in left postcentral site at the beginning of P2 and occipital and frontal ERS from the 2-second period to the end of the phase. High alpha ERD was present during the whole P2 with initial left parietal distribution spreading to left central sites at the end of the phase. **P3** presented occipital and bilateral temporal delta ERS mainly during the first half of the phase while at the end, ERD in theta and alpha rhythms were widely distributed over the scalp. **P4** presented delta ERS that moved from frontal to occipital for being largely distributed at the end of the phase, and delta and alpha ERD potentiated through time, covering the full scalp at the end of the phase.

We performed an ERS/ERD analysis of the mu rhythm (8–13 Hz) at electrodes C3, Cz, and C4, as shown in Supplementary Figure A2 (Appendix). During P1, these sites exhibited a mu ERS, which can also be seen in Fig. 6C. From P2 through the end of P4, a progressively stronger mu ERD was observed across the phases. This ERS/ERD pattern mirrors the respiratory muscle activity with an initial elastic recoil after inspiration reflected by early ERS in P1 and gradually intensifying muscle recruitment evidenced by the growing ERD across P2 to P4, highlighting the mounting engagement of the motor cortex as the phases progress.

**Fig. 7:**
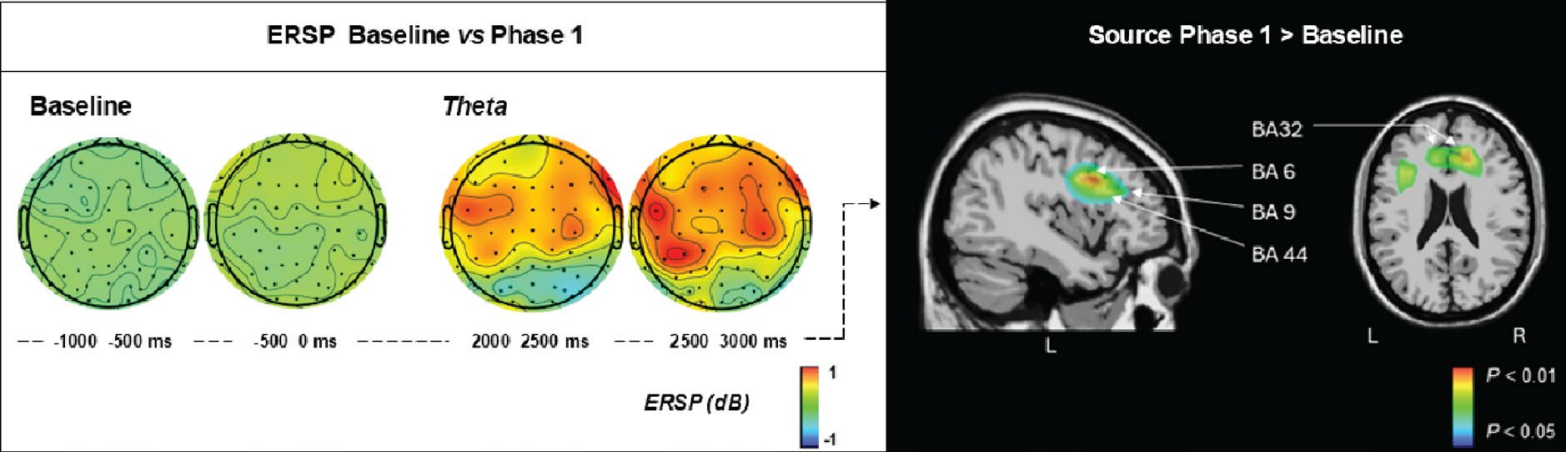
The contrast in theta rhythm generators observed during the 3rd second of phase 1 compared to baseline.

**Fig. 8:**
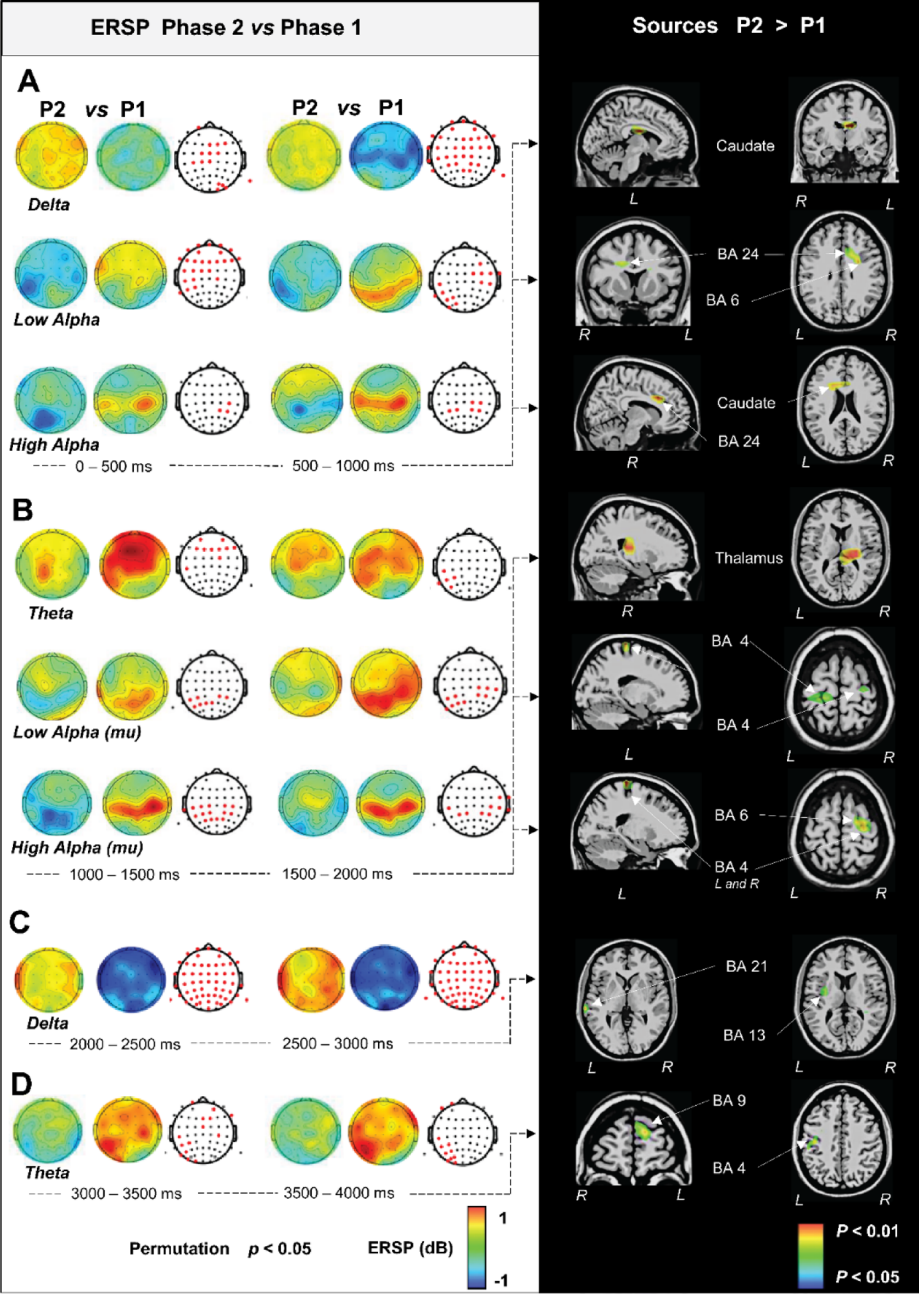
Statistically significant differences of ERSP topographical maps (in the left) and estimated generators of the ERSP topographies (in the right) for Phase 2 vs. Phase 1.

**Fig. 9:**
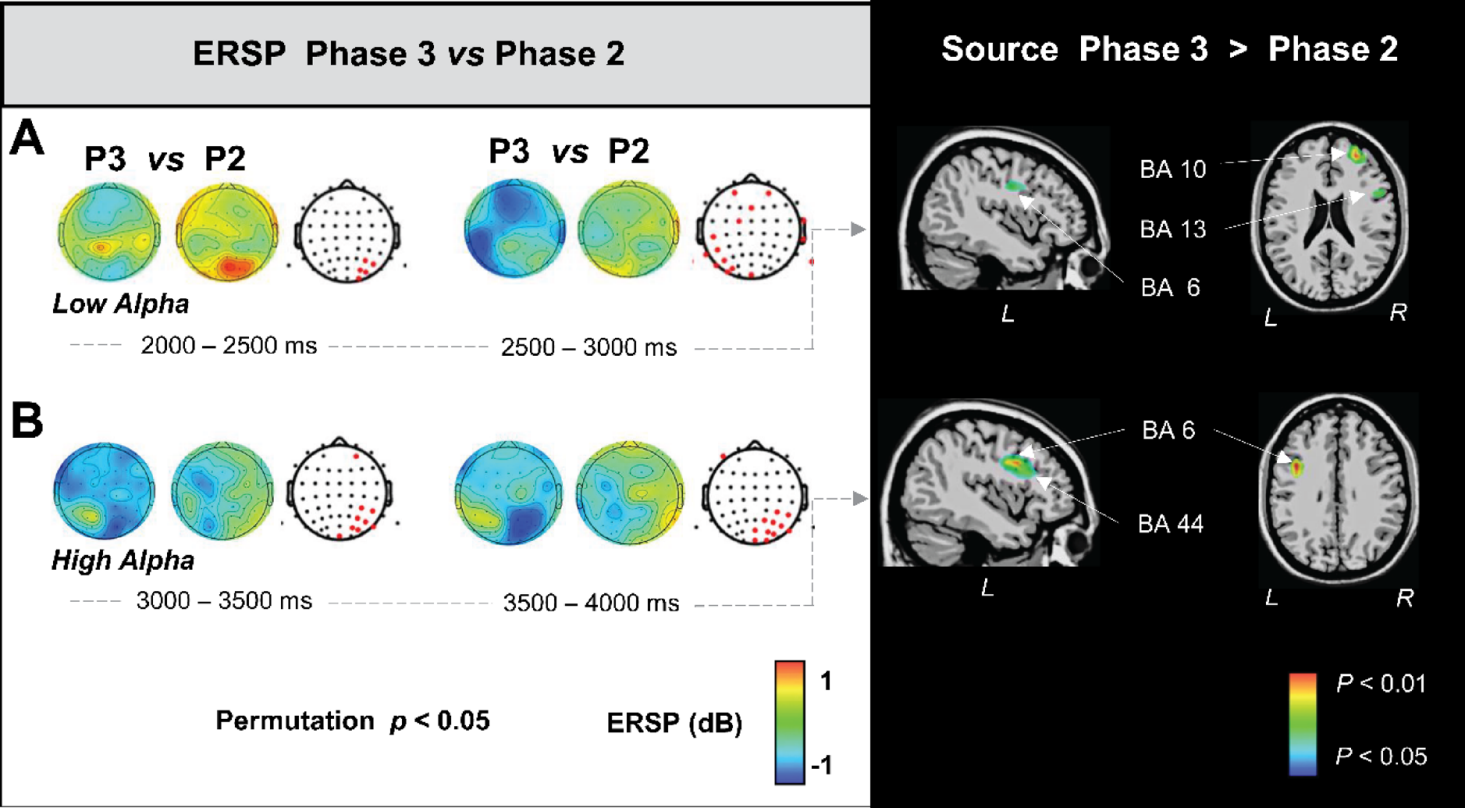
Statistically significant differences of ERSP topographical maps (in the left) and estimated generators of the ERSP topographies (in the right) for Phase 3 vs. Phase 2.

**Fig. 10:**
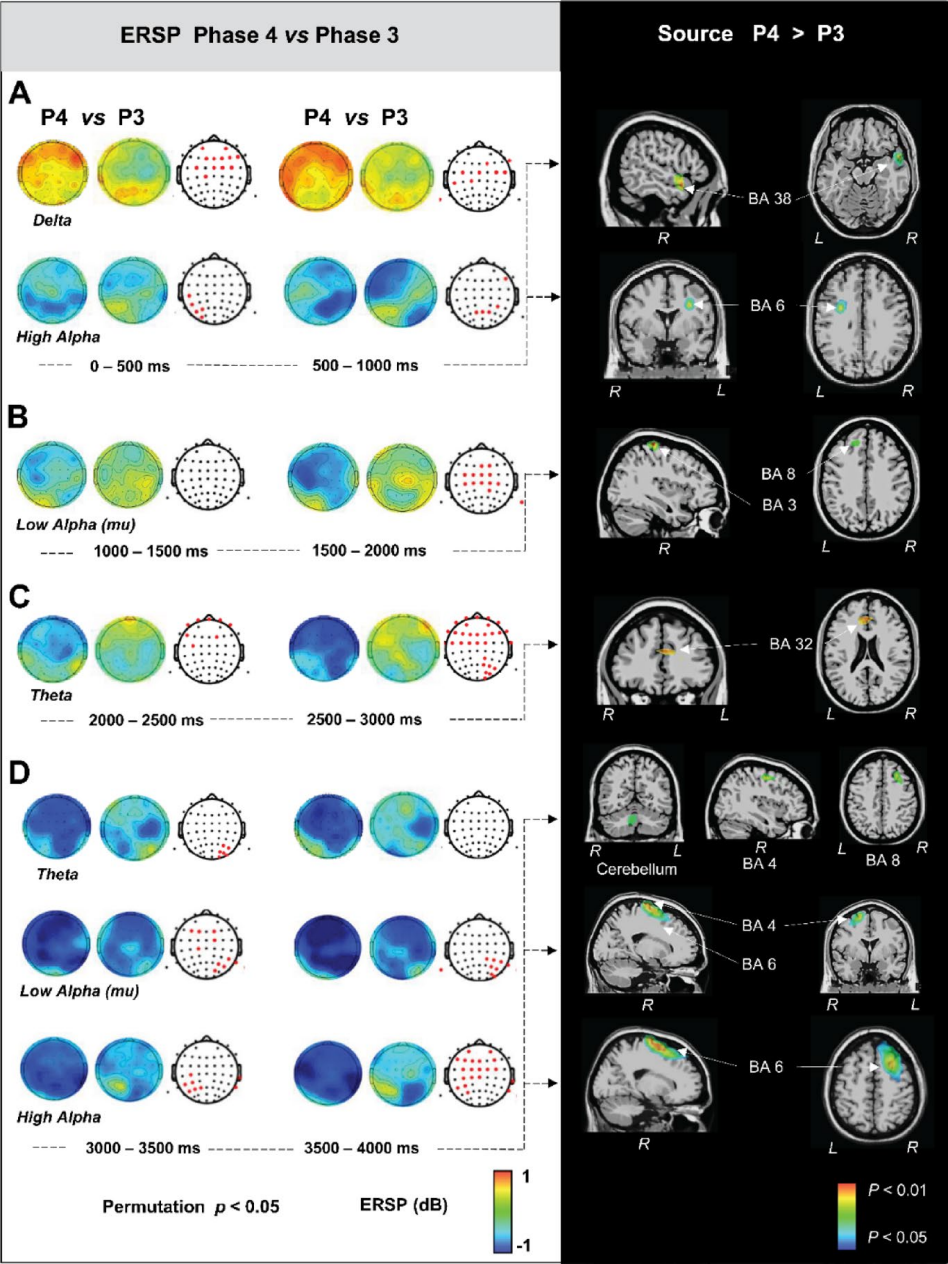
Statistically significant differences of ERSP topographical maps (on the left) and estimated generators of the ERSP topographies (on the right) for Phase 4 vs. Phase 3.

Figures 7, 8, 9 and 10 on the left, illustrates the significant differences of ERSP topographical maps between consecutive phases along phonation progression, in steps of 500 ms, for delta (1–3.5 Hz), theta (4–7 Hz), low alpha (7.5–10 Hz) and high alpha (10.5–13 Hz), respectively (the lack of image for a determined period meaning that there was not statistically significant difference for that period). Given that it is well known that at scalp level pitfalls associated with mixing of multiple cortical processes by volume conduction may occur and that consequently the inferences about the brain areas responsible of such topographical differences is not straightforward^24^. It is why we investigated the estimated generators of the ERSP topographies at the sources level by swLORETA which is a distributed solution which uses the entire brain (grey matter) volume as space or research for generators. Figures 7, 8, 9 and 10, on the right, illustrate the nonparametric statistical maps of the ERSP brain sources for each frequency band for each phase with respect to the previous one. This allowed us to highlight the brain generators accounting for the phases’ progressive transitions with the contrasts for P1 > baseline, P2 > P1, P3 > P2 and P4 > P3.

Figure 7 on the right illustrates the significant cerebral sources related to the P1 > baseline contrast, accounting for the largely extended over the scalp theta power increase (ERS, on the left). The model revealed implication of the limbic lobe bilaterally, within the anterior cingulate cortex (BA32) (14, 27, 26 and − 13, 27, 26), as well as left premotor gyrus (BA6) (-41, -4, 30), left inferior frontal gyrus (BA44), and left middle frontal gyrus (BA9) (-40, 3, 30).

Figure 8, on the left, illustrates the significant ERSP topographic differences between P1 and P2, and the underlying significant brain sources estimated by the inverse model (P2 > P1 contrast). During the first second of P2 a delta ERS across the entire scalp was observed. The source model showed that the left caudate body (Sub-lobar, Caudate) (-10, -13, 21) was involved in such power spectrum change. Simultaneously, at the scalp level a low alpha ERD was localized in the central and right frontal areas and the left parietal area. This was accompanied by the activity of the right limbic lobe’s cingulate gyrus (BA24) (16, 5, 32) and right premotor cortex (BA6) (31, -1, 31). Also, a high alpha ERS in the frontal scalp area, and an ERD in the left parietal and right central areas accounted for the involvement of right BA24 (9, 13, 31), along with the left caudate body’s (-18, 10, 23) (Fig. 8A). During the 2nd s of P2 (> P1), a theta ERD was localized in frontal and central scalp areas and were accompanied by the right pulvinar of the thalamus (Sub-lobar, Thalamus) (19, -23 12) involving a mu (low alpha) ERD in the left central and right centro-parietal scalp areas was explained by bilateral primary motor cortex (BA4) (-16, -33, 67 and 28, -21, 61). A mu (high alpha) ERD in the central scalp region was explained by bilateral motor (BA4) (27, -21, 61 and − 19–30 68) and right premotor (BA6) (26, -15, 57) (Fig. 8B). Later, at the third second the generalized delta ERD over the scalp corresponded to the left insula (BA13) (-34, -9, 13) and the left middle temporal gyrus (BA21) (-68, -28, 2) involvements (Fig. 8C). Finally, the decrease of the theta ERD observed in the scalp during the fourth second was explained by the left motor cortex (BA4) (-39, -17, 36) and the left superior frontal gyrus (BA9) (-21, 58, 27) (Fig. 8D).

Figure 9 illustrates the significant brain sources for the P3 > P2 contrast (on the right) accounting for the related significant power spectrum changes over the scalp (on the left). Significant brain sources’ implication was observed in the third second, during which right superior frontal gyrus (BA10) (25, 44, 25), right insula (BA13) (45, 6, 15) and left frontal premotor gyrus (BA6) (-43, -12, 30) accounted for a low alpha ERD situated in the left frontal, central, and parietal scalp areas (Fig. 9A). In the fourth second, the left frontal premotor cortex (BA6) (-41, 1, 30) and the left Broca’s area (BA44) involvements were observed accounting for, the high alpha ERD observed in the left frontal and right parietal scalp regions (Fig. 9B).

Figure 10 illustrates the significant brain sources for the P4 > P3 contrast (on the right) accounting for the related significant power spectrum changes over the scalp (on the left). During the first second, the right superior temporal gyrus (BA38) (55, 7, -9) involvement accounted for, the delta ERS localized in the frontal scalp region. Simultaneously, left precentral gyrus (BA6) (-34, -6, 31) accounted for high alpha ERS over the left frontal area and concomitant right parietal ERD (Fig. 10A). During the 2nd second, the right postcentral gyrus (BA3) (38, -32, 61) and the left middle frontal gyrus (BA8) (-16, 29, 38) involvement explained the low alpha ERD situated in the left frontal, central and right parietal scalp areas (Fig. 10B). In the third second, the widely extended theta ERD over the scalp was explained by the left anterior cingulate cortex (BA32) (-6, 29, 25) (Fig. 10C). Finally, in the fourth second, the generalized theta ERD over the entire scalp was accompanied by the right motor cortex (BA4) (38, -12, 48), right middle frontal gyrus (BA8) (26, 15, 42), and right anterior cerebellar nodule (9, -57, -30) involvements. The generalized mu ERD and low alpha ERD over the scalp was explained respectively by right motor cortex (BA4) (19, -21, 69) and right premotor cortex (BA6) (18, 8, 60) and the widely extended high alpha ERD over the scalp was explained by the right premotor cortex (BA 6) (18, -17, 67) (Fig. 10D).

All in all, we observed that prefrontal and premotor areas were implicated in every phase relative to the preceding one. Distinctively, P2 presented primary motor cortex involvement accompanied by subcortical thalamic and basal ganglia activity. Additionally, distinctively, P4 presented cerebellar involvement accompanying primary motor cortex activity.

## Discussion

In the present work, we investigated brain function through the power modulations of EEG brain rhythms to estimate the brain generators related to phase transitions in long utterance phonation^5^ .This specific phonation task implies a dual constraint (1) to produce the ‘pa’ syllable and (2) to use respiratory resources efficiently without resuming an inspiratory cycle.

It is currently well accepted that the pons, making part of subcortical respiratory circuitry, plays a significant role in the physiological control of breathing, and in particular for the respiratory cycles^59,60^. However, the role of the central nervous system in the voluntary component of the expiratory phase required for phonation remains to be elucidated. Correlations between EEG brain rhythms and acid base balance during voluntary breath-holding have shown complex interactions between delta and alpha rhythms, and breathing^61^. Also, the enhancement of cortical oscillations along with voluntary increase of respiratory rate, highlighted the role of the cortex in respiratory control^27,62^. It is well known that phonation cannot be dissociated from respiration, as the phonatory mechanism relies on a precise and complex coordination with the respiratory system, the so called pneumo-phonic coordination^63^. In this line, the present protocol considers events defining phases, as markers characterized by both Ps, and muscular activity as previously introduced by Ladefoged, 1967^5^.

When focusing on the power modulations of phonation phases in relation to a common reference period preceding phonation, it was observed that delta and high alpha ERD lasting throughout the four phases emerged during phonation. This seems in line with a motor program of repetitive movement, or phonation of repetitive “pa” through the long utterance. During repetitive movements, sensorimotor cortex and premotor cortex remain active to maintain coordination and fluidity of movements, and delta, alpha and beta oscillations express ERD during the movement production^64–66^.

In addition to the delta and alpha ERD, ERS of a more diffuse band between the low alpha and theta ranges emerged from the middle of P1 to the end of P3. Subsequent spectral perturbations analysis associated to each phase transition highlighted a global topographical ERS/ERD inversion involving all four oscillation bands (delta, theta, low, and high alpha), which became progressively more apparent across the different phases. This inversion was particularly noticeable when focusing on P1 and P4. Delta ERD, which was widely extended over the scalp in P1, transformed into a widely extended delta ERS in P4. Conversely, frontal, central, and parietal theta ERS, low alpha ERS, and high alpha ERS in P1 transitioned into largely extended ERDs over the scalp.

It has been previously proposed that phono-articulatory theta oscillation may be an intrinsic mechanism already present in primate vocal production^13^. Although the present study cannot resolve whether such theta rhythmicity is cortically controlled^13–18^it can corroborate that specific patterns of cortical rhythms accompany phonation in the theta range (4 Hz rhythm of “pa” production) across the four phases in long utterances.

Theta rhythm, particularly in its frontal midline component, is widely recognized as a marker of executive functions such as cognitive control, conflict monitoring, active maintenance in working memory and sustained attention in the adaptive processing of salient information (Gärtner et al. 2015; Chien et al. 2017)^67,68^.

However, theta oscillations can also be modulated by mental fatigue, as demonstrated by Wascher et al. (2014)^69^ in a prolonged Simon task. They reported a linear increase in frontal and parietal theta activity, correlated with a progressive decline in behavioral accuracy over time. In our findings, a clear theta ERS consistent with an active cognitive engagement profile is visible at the beginning of P1 in fronto-central regions, remaining stable till the end of P3. In contrast, a generalized theta ERD gradually emerges from P2 through P4, spreading across the entire scalp. Although the lack of specific measures of fatigue in our study prevents us to exclude a major role, this evolving theta pattern observed does not seem to align with the typical trajectory of progressive mental fatigue but could reflect a functional state shift^69^.

Alpha activity, likewise, presents patterns that are partially consistent with, but also divergent from, those associated with fatigue and cognitive processes.

Occipital or parietal alpha ERD is frequently observed during attentional engagement with task-relevant stimuli, reflecting a release of neuronal inhibition that facilitates perceptual or mnemonic processing (Hanslmayr et al., 2012; Bacigalupo & Luck, 2022)^70,71^. In cognitive conflict tasks, frontal alpha ERD has been associated with the activation of executive control networks (Tang et al., 2013)^72^. Conversely, alpha ERS in task-irrelevant cortical regions as reported by Rihs et al. (2007)^73^ is interpreted as a mechanism of active inhibition, contributing to attentional focus by suppressing distractors^74,75^.

Studies of physical fatigue, such as Enders et al. (2017)^76^have shown that prolonged exertion can amplify alpha ERD, reflecting an increased cortical demand to maintain performance. In our study, we observed a progressive high alpha ERD starting from the beginning till the end of the task, consistent with sustained attentional engagement in fatigue. Finally, the alternation of ERS and ERD observed from phases P2 to P4 along with the generalized alpha ERD across the scalp from P4 onward suggests a complex spatiotemporal modulation involving both motor control dynamics and those potentially related to cognitive load and emerging fatigue.

The distributed inverse solution (swLORETA) was applied to significant variations in the topographical power spectrum between consecutive phases to highlight the brain sources relevant to each phase. In this analysis, P1, compared to the preceding baseline, corresponding to the end of inspiration and the maximal peak of Ps, was characterized by the involvement of the frontal lobe. This involvement aligns with a voluntary, free-will motor execution network for phonation^77^beginning at P1. Specifically, it includes the participation of the associative prefrontal cortex (middle frontal gyrus, BA9), bilateral anterior cingulate cortex (BA32), Broca’s area (left inferior frontal gyrus, BA44), and the left precentral gyrus (BA6).

In the subsequent phase, P2, compared to P1 (P2 > P1), there was involvement of the bilateral primary motor cortex (BA4) in the mu oscillation and the right premotor cortex (BA6) in the alpha oscillations, accompanied by the bilateral anterior cingulate cortex. This suggests a subcortical-cortical interplay during P2, possibly accounting for sensorimotor monitoring expressed by the mu involvement. The left superior frontal gyrus (BA9) and the sensorimotor cortex were engaged in the theta oscillations, along with the involvement of the right thalamus. The right thalamus, together with the left caudate nucleus, showed activity in the delta and high alpha oscillations, which was the only subcortical activity observed across the four phases. This suggests a subcortical-cortical interplay during P2, possibly accounting for sensorimotor monitoring, which could be responsible for the stabilization of intra-oral pressure. This, in turn, implicates the activation of the internal intercostal muscles and the concurrent programming of subsequent external oblique muscle recruitment.

Later, P3 (P3 > P2) was characterized exclusively by the involvement of superior frontal (BA 10) and premotor (BA6) areas in the alpha oscillation which could reflect a distinctive volitional programing of phonation necessitating clear external intercostal and external oblique activation while maintaining intra-oral pressure.

P4 is characterized by an initial drop in intra-oral pressure, followed by a subsequent increase towards the end while muscular activities reach their peak. At the source level, P4 (P4 > P3) presented similarly to P2, with bilateral primary motor cortex (BA4) and bilateral premotor cortex (BA6) involvement across rhythms. Interestingly, P4 showed distinctive involvement of the right cerebellum in the theta oscillations, which was concomitant with motor cortex (BA4) and frontal cortex (BA8, BA32). This suggests that the cerebellum contributes, through its connections with BA4 and BA8^78^, to fine-tuning movement precision, muscle tone, and timing regulation, all of which are crucial for managing the intra-oral pressure drop during the finalization of the long-duration utterance.

It is important to note that the involvement of the anterior cingulate cortex was observed in P1, P2, and P4. While it may underly the selection and maintenance of attentional and physical activity resources in P1 and P2, ensuring that phonation continues with appropriate effort throughout the long utterance and appropriates motor patterns, it is possible that in P4, the anterior cingulate cortex was also responsible for sustaining persistence and focus, allowing the participants to complete the phonation until exhaustion^79^.

The implication of the delta oscillation localized in the insula (left BA 13) and temporal lobe (left BA21, right BA38) in P2 and P4 respectively, could reflect key processes in perceptual stabilization and the prevention of verbal transformations, related to the repeated production of the syllable [pa]. The left BA13 region is involved in directed attention management and auditory perceptual processing, facilitating the maintenance of the repeated syllabic form and minimizing spontaneous perceptual deviations, likely through reinforcement of temporary auditory memory^80–83^. Additionally, the involvement of the left BA21, a region of the middle temporal gyrus associated with phonetic and semantic integration, suggests that sustained activation in this area could reduce “phonetic satiation” (satiation effect), which often leads to verbal transformations^84^. This phenomenon could thus be modulated by active repetition, stabilizing phonetic representations over time and preventing undesirable reorganizations (e.g., saying “Papa” instead of “pa”). Finally, the activation of the right BA38, located in the right anterior temporal cortex, could contribute to the integration and semantic consolidation of the perceived syllabic form^84^. This process would help stabilize auditory interpretation by synchronizing contextual memory with immediate perception. Delta oscillation in these areas supports the hypothesis of a slow perceptual consolidation network, essential for inhibiting spontaneous verbal transformations in a continuous repetition task^85^. By reinforcing the integrity of the initial auditory representation, these activations effectively prevent phonetic variations within this repetitive task^86,87^.

The results of the present study highlight the involvement of oscillatory dynamics and their cortical-subcortical sources accompanying the respiratory muscular patterns through differentiated phases during a prolonged phonatory action. By placing the participant in a situation where they must maintain continuous vocalization without taking another breath, our paradigm introduces a psycho-physiological conflict, in which the preservation of internal homeostasis is confronted with a demanding behavioral instruction. From a neurophysiological perspective, this situation sheds light on the brain’s ability to anticipate, coordinate, and finely adjust ventilatory commands based on sensory feedback (intra-oral pressure, internal feedback). Together, these psycho-physiological and neurophysiological dimensions align with a holistic approach to respiratory control during phonation, paving the way for innovative multidimensional therapeutic interventions^88,89^.

Building on these findings, our results also invite further exploration of the role of oscillatory brain dynamics in natural speech production. Speech, as a complex motor activity, requires precise coordination between breathing, phonation, and articulation. The observed contribution of brain oscillations to the control of respiratory muscles under constraint suggests that these rhythms may play a central role in other aspects of vocal production, such as the articulation of complex word sequences, phonemic transitions, and prosodic modulation. Understanding the interaction between oscillatory brain activity and sensorimotor networks could help identify specific neurophysiological signatures that reflect the strategies underlying fluent and intelligible speech production. Moreover, extending this work to faster oscillatory bands, particularly the beta rhythm (13–30 Hz), could illuminate the fine sensorimotor integration^25,26^.

This perspective opens avenues for developing integrative models of speech production that combine brain activity measurements with analyses of articulatory and aerodynamic mechanisms. Such interdisciplinary approaches could have far-reaching applications, including speech rehabilitation following neurological disorders, advancements in speech synthesis technologies, and improvements in language learning strategies^75^. In this context, the investigation of oscillatory brain dynamics in respiratory control during speech represents a promising research direction for better understanding and modeling the intricate relationships between brain activity, respiration, and vocal production.

Furthermore, we believe that the present findings could be particularly relevant in the context of Parkinsonian dysarthria, where impairments in respiratory-phonatory coordination are frequently observed (Ponchard et al., 2023)^90^. Future studies should investigate whether individuals with Parkinson’s disease exhibit the same four phases of phonation identified in our study. If not, it would be important to determine at which phase(s) the breakdown occurs and to characterize the associated cortical patterns. Such findings could contribute to the development of targeted clinical interventions, including biofeedback-based therapies or diagnostic tools tailored to specific speech motor deficits.

## Limitations and methodological considerations

The use of the syllable [pa] in our paradigm was a deliberate methodological choice, grounded in both articulatory simplicity and physiological relevance. The bilabial plosive [p] involves complete closure of the lips, leading to a build-up of intraoral pressure that is released during the production of the vowel [a]. This configuration provides a reliable, non-invasive approximation of subglottic pressure (Ps), as the glottis remains open during [p], allowing the pressure to reflect the total volume of the lungs and vocal tract. Compared to other speech sounds that may be affected by nasalization or complex tongue movements, [pa] is relatively easy to produce and highly reproducible across trials and participants. This consistency is essential for tracking Ps dynamics over time, especially in a task requiring continuous phonation without breathing.

Although the [pa] train is not representative of natural speech, similar respiratory and phonatory mechanisms are engaged during extended utterances in natural contexts, such as counting or reading aloud for more than 4–5 s (Ladefoged et al., 1967). Moreover, the estimation of Ps is critical in speech production, as it must remain quasi-constant throughout an utterance (Ohala, 1970; Collier, 1975; Fant et al., 2000; Demolin, 2006)^91–93^. The paradigm also draws parallels with real-life situations requiring sustained phonation, such as theatrical performance, public speaking, singing, and liturgical chanting (e.g., OM chanting), where vocal control must be maintained beyond the elastic recoil of the thoracic cage.

To ensure that the task did not introduce confounding artifacts, we used a specially designed mouthpiece that does not interfere with sound production or introduce resonance effects. Additionally, no nasal airflow was involved, and any vocal fold adjustments that could have affected phonation would have been detected through changes in F0 or electroglottographic (EGG) recordings—none of which were observed. Trials with undesirable movements (e.g., torso shifts, yawning) were systematically excluded. This methodological rigor supports the validity of our approach and reinforces the relevance of [pa] as a controlled yet physiologically meaningful stimulus for investigating subglottic pressure regulation and its cortical correlates.

Our study used source reconstruction methods in order to address the pitfalls at the scalp level due to the mixing of multiple cortical processes through volume conduction (Makeig et al., 2004), which makes it impossible to infer and assess the involved generators accounting for the observed power spectral variations related to the specific phase transitions.

Reconstruction models have limitations per se, as they rely on priors to ensure the uniqueness of the solution. Nevertheless, source reconstruction from EEG signals remains relevant because, unlike fMRI, EEG provides a direct measure of the (global) real electrical activity of the brain. The choice of a distributed solution, instead of a discrete one, allowed us to avoid a restrained space for the generator search.

Among swLORETA’s limitations are the spatial resolution and source spread (around 14 mm, Benjamin C. Cox et al., 2025)^94^and its risk of false positives (“ghost sources”), especially when deep and cortical activity are simultaneous (Attal, Y. et al., 2013)^95^. However, in simulated data, it has been shown that swLORETA enabled the accurate reconstruction of surface and deep current sources even in the presence of noise and when two dipoles are simultaneously active. This was achieved by incorporating a singular value decomposition-based lead field weighting that compensates for the varying sensitivity of the sensors to current sources at different depths (Palmero-Soler et al., 2007; Palmero-Soler, 2016)^96,97^. Although the contribution of subcortical structures to EEG remains debated, direct evidence has shown that scalp EEG is indeed capable of detecting (and correctly localizing via distributed source reconstruction) signals recorded from intracranial electrodes placed in the Centro medial thalamus and the nucleus accumbens (Seeber M. et al., 2019)^98^. In this context, deep sources located in the cerebellum have previously been reported through inverse solution models (Reyes et al., 2005; Elshoff et al., 2013; Stancak and Fallon, 2013; Proverbio et al., 2014, 2017; Cebolla et al., 2016, 2022)^99–105^.

## Conclusion

This study highlights the complex oscillatory brain patterns underlying the coupling among respiratory control, phonatory production and the entailed perceptual maintenance of the repeated syllable, where activity, and Power modulations of delta, theta, and alpha brain oscillations indicate a progressive reorganization of neural networks involving frontal, motor, cingulate, temporal, insular, and cerebellar areas. Taken together, these mechanisms appear to allow continuous adaptation or monitoring, ensuring not only expiratory efficiency and motor precision but also the perceptual stability of repeated vocal action. These findings pave the way for future research into the generators of cerebral rhythms and the rapid dynamics of brain activity involved in phonation control, leveraging the high temporal resolution of EEG.

## Supplementary Information

Below is the link to the electronic supplementary material.

Supplementary Material 1

## Data Availability

The datasets generated during and/or analyzed during the current study are available from the corresponding author upon reasonable request.
