# Supplementary material for: EEG oscillations and related brain generators of phonation phases in long utterances
Source: Sci Rep. 2025 Aug 9;15:29150. doi: 10.1038/s41598-025-13901-8 (PMC12335572; doi:10.1038/s41598-025-13901-8)
Supplement: Supplementary file 1 — Supplementary Material 1 [file 41598_2025_13901_MOESM1_ESM.pdf]

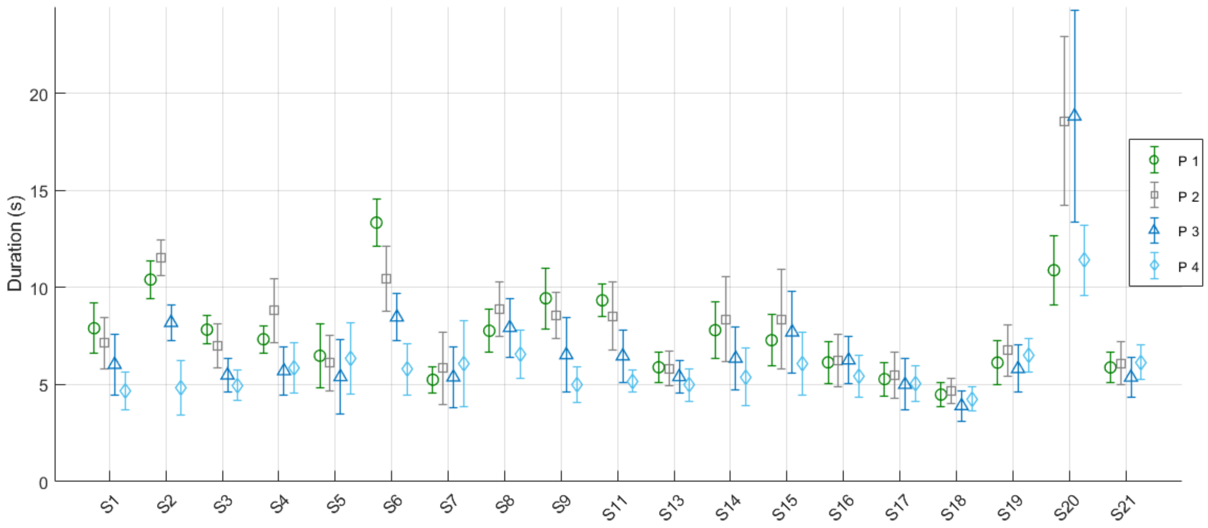

Figure A1: Intra-subject variability in phase duration. P: phase, S: subject. Circles, squares, triangles, and diamonds represent the mean duration of phases 1 to 4, respectively.

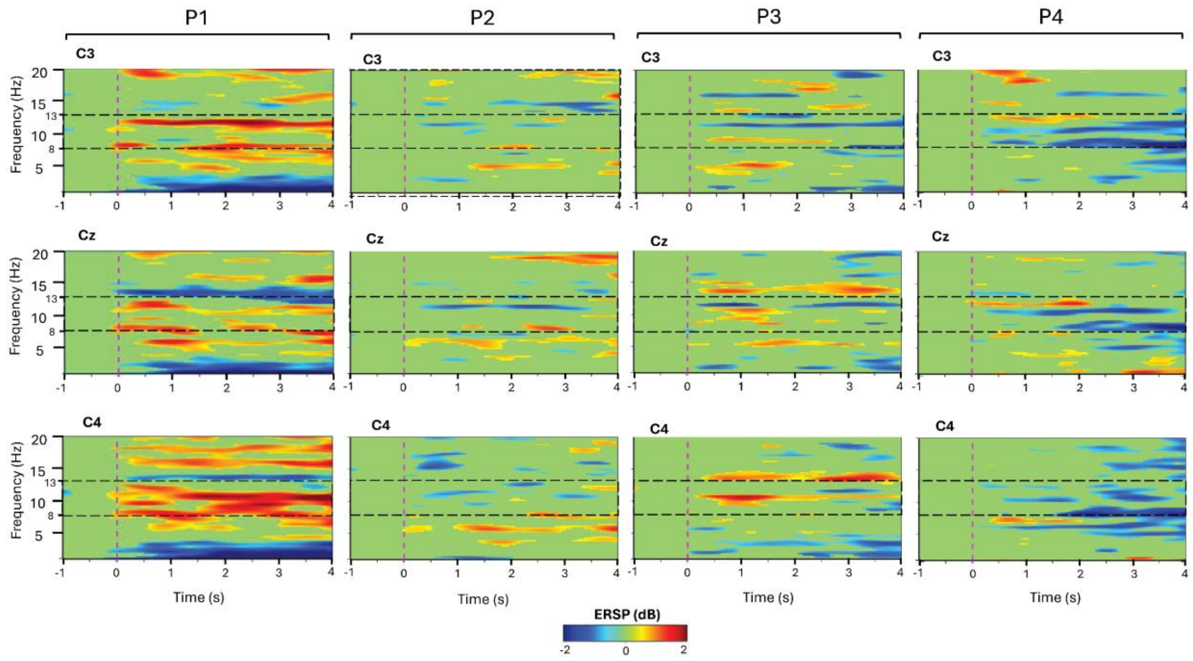

Figure A2: Grand average (bootstrap $p < 0.001$ FDR) of the power spectral variations of the EEG oscillations during phonation. ERSP templates (C3, Cz, C4) for each phase. The dashed boxes indicate the boundaries of the 8-13Hz band (mu rhythm), P: phase.

Table A1. Summary table of intra-subject variability in phase duration. P: phase, S: subject, std: standard deviation. Each row corresponds to a subject; values represent the duration of each phase across trials, in seconds.

Subject	Mean P1	std P1	Mean P2	std P2	Mean P3	std P3	Mean P4	std P4
**S1**	7.90	1.30	7.14	1.30	6.03	1.56	4.67	0.95
**S2**	10.41	0.96	11.53	0.91	8.19	0.94	4.84	1.41
**S3**	7.83	0.74	7.01	1.13	5.48	0.84	4.96	0.79
**S4**	7.34	0.70	8.81	1.63	5.70	1.25	5.87	1.30
**S5**	6.49	1.63	6.11	1.43	5.40	1.94	6.33	1.84
**S6**	13.35	1.19	10.46	1.68	8.47	1.20	5.79	1.32
**S7**	5.25	0.67	5.84	1.88	5.38	1.58	6.09	2.23
**S8**	7.77	1.10	8.90	1.41	7.93	1.52	6.57	1.23
**S9**	9.45	1.57	8.56	1.21	6.53	1.93	4.99	0.93
**S11**	9.34	0.85	8.51	1.75	6.47	1.35	5.18	0.56
**S13**	5.90	0.79	5.83	0.89	5.40	0.82	4.98	0.83
**S14**	7.80	1.46	8.37	2.18	6.35	1.64	5.39	1.50
**S15**	7.29	1.31	8.36	2.56	7.70	2.09	6.09	1.62
**S16**	6.14	1.08	6.24	1.37	6.26	1.21	5.43	1.09
**S17**	5.28	0.86	5.48	1.18	5.00	1.32	5.05	0.94
**S18**	4.49	0.60	4.68	0.64	3.90	0.79	4.26	0.62
**S19**	6.13	1.15	6.76	1.32	5.82	1.21	6.51	0.86
**S20**	10.90	1.78	18.58	4.34	18.83	5.43	11.41	1.79
**S21**	5.88	0.77	6.10	1.11	5.37	1.05	6.16	0.91
